# Antimicrobial prophylaxis administration after umbilical cord clamping in cesarean section and the risk of surgical site infection: a cohort study with 55,901 patients

**DOI:** 10.1186/s13756-020-00860-0

**Published:** 2020-12-22

**Authors:** Rami Sommerstein, Jonas Marschall, Andrew Atkinson, Daniel Surbek, Maria Gloria Dominguez-Bello, Nicolas Troillet, Andreas F. Widmer, Swissnoso, Carlo Balmelli, Marie-Christine Eisenring, Stephan Harbarth, Jonas Marschall, Didier Pittet, Hugo Sax, Matthias Schlegel, Alexander Schweiger, Laurence Senn, Nicolas Troillet, Andreas F. Widmer, Giorgio Zanetti

**Affiliations:** 1 Department of Infectious Diseases, Bern University Hospital, University of Bern, Freiburgstrasse, 3010 Bern, Switzerland; 2 Swissnoso, National Center for Infection Control, Bern, Switzerland; 3 Department of Obstetrics and Gynaecology, Bern University Hospital, University of Bern, Bern, Switzerland; 4 Swiss Society of Obstetrics and Gynaecology, Bern, Switzerland; 5 Department of Biochemistry and Microbiology, Rutgers School of Environmental and Biological Science, New Brunswick, NJ USA; 6 Service of Infectious Diseases, Central Institute, Valais Hospitals, Sion, Switzerland; 7 Department of Infectious Diseases, University Hospital Basel, Basel, Switzerland

**Keywords:** Cesarean section, Microbiome, Modelling, Obstetrics, Surgical antimicrobial prophylaxis, Surgical site infection

## Abstract

**Background:**

The World Health Organization (WHO) recommends administration of surgical antimicrobial prophylaxis (SAP) in cesarean section prior to incision to prevent surgical site infections (SSI). This study aimed to determine whether SAP administration following cord clamping confers an increased SSI risk to the mother.

**Methods:**

Study design: Cohort. Setting: 75 participating Swiss hospitals, from 2009 to 2018. Participants: A total of 55,901 patients were analyzed. Main outcome measures: We assessed the association between SAP administration relative to incision and clamping and the SSI rate, using generalized linear multilevel models, adjusted for patient characteristics, procedural variables, and health-care system factors.

**Results:**

SAP was administered before incision in 26′405 patients (47.2%) and after clamping in 29,496 patients (52.8%). Overall 846 SSIs were documented, of which 379 (1.6% [95% CI, 1.4–1.8%]) occurred before incision and 449 (1.7% [1.5–1.9%]) after clamping (*p* = 0.759). The adjusted odds ratio for SAP administration after clamping was not significantly associated with an increased SSI rate (1.14, 95% CI 0.96–1.36; *p* = 0.144) when compared to before incision. Supplementary and subgroup analyses supported these main results.

**Conclusions:**

This study did not confirm an increased SSI risk for the mother in cesarean section if SAP is given after umbilical cord clamping compared to before incision.

## Manuscript at a glance

**Why was this study conducted?** This study is by far the largest study to assess the association between timing of surgical antimicrobial prophylaxis relative to cord clamping and surgical site infection in cesarean section patients.

**What are the key findings?** We were able to demonstrate that the surgical site infection rate is not higher if the prophylaxis is administered after cord clamping. The same surveillance method was used over many years. The drop-out rate was low and the quality of the data very high due to a rigorous validation system.

**What does this study add to what is already known?** The results of this large prospective study provide evidence that the risk of surgical site infection for the mother in cesarean section is not increased if antimicrobial prophylaxis is given after umbilical cord clamping. This could be beneficial for the neonate’s developing microbiome.

## Introduction

Surgical antimicrobial prophylaxis (SAP) for cesarean section provides a 60–70% reduction in postpartum endometritis and a 30–65% reduction in wound infections in women who undergo either elective or emergent cesarean delivery [1, 2]. The practice of administration of antimicrobials in cesarean delivery after umbilical cord clamping was common until 2013 [3]. The principal reasons were to avoid exposure to betalactam antibiotics that interfere with Vitamin K, in particular moxalactam, leading to serious bleeding [4], and to not expose the newborn to other potential side effects of antibiotics so early in life.

In addition, antimicrobials could theoretically promote the selection of resistant organisms and potentially mask neonatal infection, complicating evaluation for neonatal sepsis [3]. Microbiome building is important for the children’s development. It is modulated and shaped by antibiotic treatment, the birth delivery mode and breastfeeding [5–9]. One example is the strong association between childhood antibiotic exposure and incidence of Crohn’s disease [10].

The results of four meta-analyses concluded that administration of SAP before surgical incision significantly reduces the risk of surgical site infection (SSI), leading to a change in the previous policy [11–14]. The latest systematic review including 6250 women with SAP after clamping versus administration prior to incision reported a 38% risk reduction of SSI [14]. Therefore, current WHO recommendations support the administration of pre-incisional SAP for cesarean section procedures [15], based in part on a Cochrane review published in 2014 [2]. Switzerland, as many other countries, changed its guidelines following the publication of evidence mentioned above, and therefore obstetricians started to increasingly administer SAP before clamping around the year 2012 [16, 17]. However, the review had limitations as it included studies from low-income as well as high-income countries and did not exclude patients with pre-existing infections [14]. Also, the absolute reduction in events of the composite infectious morbidity was low.

Recent data, including a large randomized trial, from general and cardiac surgery showed that administration of SAP very close to incision was not associated with an increased risk of SSI [18, 19]. The time interval between incision and cord clamping is around 2–8 min [20], a very short period to assume a huge difference in the risk of SSI between the two strategies.

In view of this, our aim was to estimate the additional risk for SSI, if SAP is given before incision versus after umbilical clamping in a large cohort. We used data generated from the Swiss SSI surveillance program, which includes on-site quality visits, uses standardized infection definitions, and is validated by board certified infectious diseases physicians [21, 22].

## Patients and methods

### Study design and setting

This is a multicenter analytic study of prospectively collected data from the Swiss national SSI surveillance program [22]. We included data from 75 healthcare institutions in Switzerland between April 2009 and December 2018. Each participating hospital records surveillance data on three different intervention types during a selected period, and then includes all consecutive patients. The surveillance includes data collection at discharge as well as rigorous post-discharge surveillance 30 days after the intervention with additional chart review in case of suspected infection. All patients were contacted at least five times by employees from infection control before being considered “lost to follow-up”. Follow-up of routine post-discharge surveillance was > 89%. Staff members of the surveillance team periodically performed on-site audits to check data quality, as published elsewhere [21, 22]. Data were finally entered in the national database.

### Participants

**Fig. 1:**
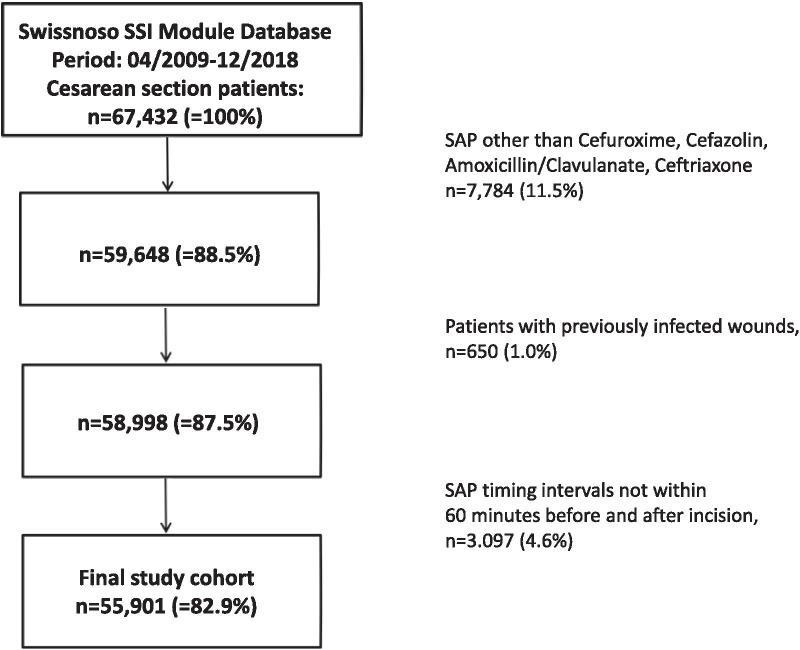
Flowchart of patient inclusion. Abbreviations: SAP: Surgical antimicrobial prophylaxis, SSI: Surgical site infection

Inclusion criteria was participation in the surveillance program and undergoing cesarean section between 2009 and 2018. Exclusion criteria were patients with preexisting maternal infections, if SAP was not applied within 60 min before/after incision, or if the single SAP agent was not cefuroxime, cefazolin, amoxicillin/clavulanate, or ceftriaxone. (Fig. 1).

### Variables, outcomes, and data sources

The primary outcome was SSI (wound infection and/or postpartum endometritis), stratified for SAP administration before incision or after clamping. Co-variables included age, Body mass index (BMI), American Society of Anesthesiologists (ASA) score, wound contamination class: clean-contaminated (standard for cesarean section) vs. contaminated (preterm rupture of the membrane without maternal signs of infection), year of surgery, SAP agent, emergent procedure, operation duration, hospital, and hospital bed-size. Minutes between SAP administration and incision were summarized by SAP administration groups. The decision of SAP administration before incision/after clamping was in most cases decided at the level of the institution. In some institutions, however, this was also a gynecologist’s decision. We accounted for these factors, by adjusting for center (clustering) and hospital size.

SSI cases were defined as patients with SSI according to Centers for Disease Control and Prevention (CDC) definitions [23]. (https://www.swissnoso.ch/module/ssi-surveillance/ueber-ssi-surveillance/das-modul/). Infection control specialists reviewed all patient data, and those patients with a suspected SSI were crosschecked by a dedicated physician. All supervising physicians—the majority board-certified in infectious diseases - had attended a training course on SSI surveillance.

Data were electronically entered into a centralized database. Type of SSI—superficial incisional, deep incisional or endometritis was recorded, as well as the pathogen (if available): Up to three different pathogens could be entered for each SSI. Clinicians were asked to enter the main causative pathogen as “first” pathogen, in cases where multiple organisms were identified. The data source for the variables was the *Swissnoso* SSI surveillance program. Primary data was obtained from the patient charts and telephone interviews with patients.

To analyze the influence of preoperative comorbidity, ASA scores were grouped into 1, 2, and high score (3–5). Age was grouped into < 30, 30–40, and > 40 years. Regarding bed size, hospitals were grouped into < 200 beds, 200–500 beds and > 500 beds.

### Data reporting

According to current *Swissnoso* data regulations, the primary data cannot be made available in a public registry [24].

### Statistical analysis

To investigate differences in terms of baseline characteristics for those with SAP before incision and after clamping we used the χ2 or Wilcoxon tests. Unadjusted general additive models (GAM) were used to visualize the SSI rate relative to timing of surgical incision using the *mgcv* package / *gam* function in R [25]. To determine the effect of SAP administration before/after clamping on SSIs, covariate adjusted multilevel logistic regression models clustering at the hospital level (random intercept) were fitted.

Two supplementary analyses were performed: for timing, periods relative to incision were grouped into different SAP timing windows (− 60 to − 30, − 29 to − 20, − 19 to − 10 [reference, as most SAP administrations before incisions were performed in this window], − 9 to incision; incision to 9, 10 to 20, 20 to 30, and 30 to 60). Secondary endpoints were the SSI outcomes superficial wound infections and deep infections (including deep wound infections and/or endometritis).

Several subgroup analyses were performed: First, two subgroup analyses were performed to ensure that administration of SAP after incision actually corresponded to administration after clamping, i.e., within 2–8 min [20]: For this reason, we first excluded cases with SAP administration in the 10 min after incision. The second subgroup excluded hospitals with variable SAP administrations before/after clamping. For this reason, SAP administration of every single institution reporting their data was screened. If > 90% of SAP administrations were performed either before or after incision, it was assumed that the institutions administered SAP consistently in the respective way and the cases were included in the subgroup analysis. Further subgroup analyses excluded patients with no information on BMI, as this variable was not mandatory and was collected in only 40% of all patients.

Missing data (including loss of follow-up) was investigated by multiply imputing data assuming missingness was at random, using the *MICE* package in R [26].

A *p-*value < 0.05 was considered statistically significant throughout. All statistics and plots were created in R [25].

## Results

**Fig. 2:**
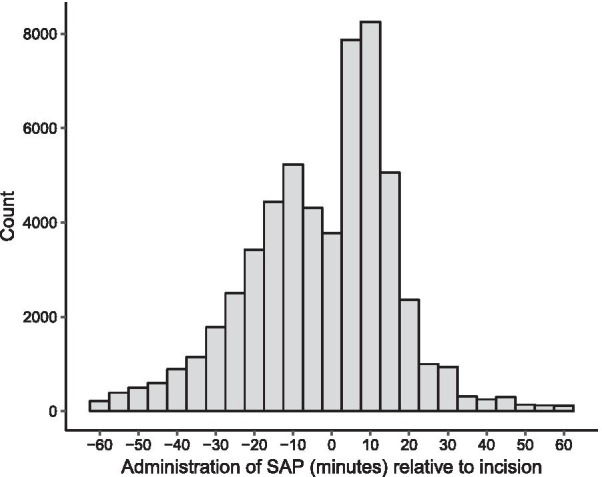
Histogram of Surgical Antimicrobial Prophylaxis Administration Relative to Incision in Cesarean Section

Out of a total of 67,432 patients, 55,901 (82.9%) fulfilled the inclusion criteria (Fig. 1). SAP was administered before incision in 26′405 patients (47.2%) and after incision in 29,496 patients (52.8%). The histogram of SAP timing relative to incision demonstrates that peak administration after incision is slightly delayed, compatible with administration after clamping (Fig. 2) and reflecting the time interval between incision and cord clamping, which usually is well below 10 min [20]. The median age of the participants was 33 years [IQR 29–36] and in 21.8% the wound class was considered contaminated. Certain differences in baseline variables were observed between the groups (Table 1). After 2014, more patients received SAP before incision, whereas large hospitals (> 500 beds) and ceftriaxone as SAP agent were more frequent in the after clamping group.

**Table 1:** Baseline and Procedural Characteristics, by Surgical Antimicrobial Prophylaxis Administration (Before Incision / After Cord Clamping)

	Before incision	After cord clamping	p
n	26,405	29,496	
Age (%)			0.043
< 30 years	7891 (29.9)	8902 (30.2)	
30–40 years	16,631 (63.0)	18,346 (62.2)	
> 40 years	1883 (7.1)	2248 (7.6)	
BMI in kg/m^2,^ median [IQR])^a^	28.0 [25.2, 31.6]	27.3 [24.2, 30.9]	< 0.001
SAP agent (%)			< 0.001
Amoxicillin + Clavulanate	3601 (13.6)	1770 (6.0)	
Cefazolin	9081 (34.4)	9073 (30.8)	
Ceftriaxone	782 (3.0)	5117 (17.3)	
Cefuroxime	12,941 (49.0)	13,536 (45.9)	
Minutes between SAP administration and incision	−15 [−25, −9]	10 [5, 15]	< 0.001
Elective surgery = yes (%)	13,458 (51.0)	14,820 (50.2)	0.089
ASA Score (%)			< 0.001
ASA 1	4427 (16.8)	6862 (23.3)	
ASA 2	20,229 (76.6)	20,648 (70.0)	
ASA 3/4/5	1401 (5.3)	1514 (5.1)	
NA	348 (1.3)	472 (1.6)	
Contaminated wound = yes (%)	5724 (21.7)	6440 (21.8)	0.663
Procedure duration in minutes (median [IQR])^b^	38 [29, 49]	37 [29, 48]	< 0.001
Hospital bed size (%)			< 0.001
< 200	12,328 (46.7)	17,588 (59.6)	
200–499	11,497 (43.5)	5757 (19.5)	
500+	2580 (9.8)	6151 (20.9)	
Procedure after January 1, 2014 = yes (%)	18,081 (68.5)	9612 (32.6)	< 0.001

*Abbreviations*

*ASA* American Society of Anesthesiologists

*BMI* Body mass index

*IQR* Interquartile range

*SAP* Surgical antimicrobial prophylaxis

^a^BMI NA: Before =15′510 (58.7%); After = 18′052 (61.2%)

^b^Duration NA: Before =144 (0.5%); After = 94 (0.3%)

Overall, 846 SSIs were found. The majority (550, 65.0%) were superficial wound infections, followed by organ space infections (endometritis; 226, 26.7%) and deep wound infections (70, 8.3%). 379 (1.6%) of patients with SAP before the incision had SSI, compared to 449 (1.7%) of patients with SAP after clamping (*p* = 0.759).

There were no differences in the SSI rates between the two groups, stratified for the depth of infection (Additional file 1: Table 1).

**Fig. 3:**
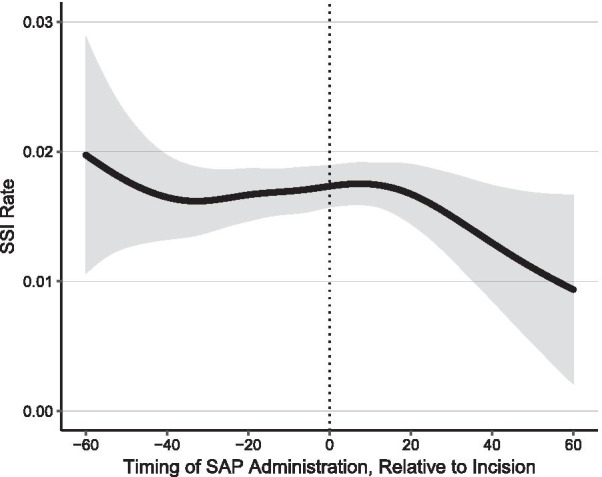
Unadjusted Generalized Additive Model with Surgical Site Infection as the Dependent Variable and Timing Relative to Incision as the Predicting Variable. The predicted SSI rate including 95% confidence intervals for the risk of surgical site infection relative to timing of SAP administration is shown. The timepoint of incision is indicated with a dashed line. Abbreviations: SAP: Surgical antimicrobial prophylaxis, SSI: Surgical Site Infection

The visual results from fitting an unadjusted GAM showed that SSI risk was stable between − 40 min to 15 min after the incision. Between − 60 to − 40 min, as well as between 15 and 60 min relative to incision, the results suggest non-significant oscillation of the SSI risk (Fig. 3) and crude SSI rates for time windows were comparable.

In the adjusted multilevel model SAP administration after clamping was not significantly associated with an increased SSI rate (OR 1.14, 95% CI 0.96–1.36; *p* = 0.144). Co-variables independently associated with an increased risk of a SSI were contaminated wound class (OR 1.27, 95% CI 1.07–1.50; *p* = 0.005), ASA score of 2 (compared to ASA score of 1; OR 1.22, 95% CI 1.00–1.48 *p* = 0.046), and increasing procedure duration (per 30 min; OR 1.33, 95% CI 1.19–1.49; *p* < 0.001).

Age between 30 and 40 years (compared to < 30 years; OR 0.76, 95% CI 0.66–0.88; p < 0.001), and elective (vs. emergent) surgery (OR 0.57, 95% CI 0.49–0.68; p < 0.001) were associated with a decreased SSI risk (Table 2).

**Table 2:** Adjusted Mixed Effects Logistic Regression Model^a^ with Surgical Site Infection as the Dependent Variable

	OR	LL	UL	p
SAP administration after cord clamping (Ref = before incision)	1.140	0.956	1.360	0.144
Age group				
30–40 years (Ref = < 30 years)	0.760	0.657	0.879	< 0.001
> 40 years (Ref = < 30 years)	0.852	0.640	1.134	0.272
Contaminated wound (Ref = clean, contaminated)	1.270	1.074	1.503	0.005
SAP agent (%)				
Cefazolin (Ref = Amoxicillin/clavulanate)	1.106	0.721	1.697	0.645
Ceftriaxone (Ref = Amoxicillin/clavulanate)	0.939	0.544	1.619	0.821
Cefuroxime (Ref = Amoxicillin/clavulanate)	1.031	0.687	1.547	0.882
Elective surgery (Ref = emergent)	0.574	0.488	0.676	< 0.001
ASA Score				
ASA 2 (Ref = ASA 1)	1.217	1.003	1.477	0.046
ASA 3/4/5 (Ref = ASA 1)	1.326	0.952	1.846	0.095
Procedure duration (per 30 min increase)	1.330	1.189	1.488	< 0.001
Hospital bed size				
200–499 (Ref = < 200)	1.200	0.880	1.634	0.249
500+ (Ref = < 200)	1.231	0.819	1.849	0.317
Procedure after Jan 2014 (Ref = before)	0.852	0.711	1.020	0.081

*Abbreviations*

*ASA* American Society of Anesthesiologists

*LL* Lower limit of 95% confidence interval

*OR* Odds Ratio

*SAP* Surgical antimicrobial prophylaxis

*UL* Upper limit of 95% confidence interval

^a^Complete cases only, *n* = 48,995

In the analysis of the 19,923 patients in which data on BMI was recorded and all further variables were available, the results were comparable with the main results and the proportion of SSI was similar at 1.85% (95% CI, 1.67–2.05). However, increasing BMI was significantly associated with SSI risk (p < 0.001; Additional file 1: Table 2).

In the supplementary analyses for the secondary outcomes, SAP administration after clamping was not significantly associated with an increased risk of either superficial wound infections (adjusted OR 1.16, 95% CI 0.93–1.44; *p* = 0.180) or the combined endpoint deep wound infections and endometritis (adjusted OR 1.07, 95% CI 0.80–1.42 *p* = 0.661, Suppl Tables 3–4).

This remained unchanged for the patients with data on BMI (Additional file 1: Tables 5–6).

An adjusted supplementary analysis, with different timing windows, was unable to identify a specific timing window associated with the SSI outcome (Additional file 1: Table 7).

Next, we aimed at verifying that administration after incision is a reliable surrogate marker for administration after clamping. In the subgroup of the 15 hospitals (15,855 patients) that administered SAP in > 90% of procedures per hospital either before or after incision the adjusted OR of SSI risk for SAP administration after clamping was 0.84 (95% CI 0.54–1.30, *p* = 0.435), in line with the main analysis (Additional file 1: Table 8). Even when excluding the 17′205 patients that received SAP within 10 min after incision, the point estimates of this conservative approach lay above 1, but there was still no significant association (adjusted OR 1.10, 0.87–1.38, *p* = 0.420; Additional file 1: Table 9).

A stratified analysis (data not shown) demonstrated that for neither of the periods (before/after 2014) SSI was significantly associated with SAP administration pre incision/after clamping.

Staphylococci, Enterobacteriaceae, anaerobes, enterococci and streptococci were the most frequently identified causative microorganisms of SSI. Supplementary Table 10 provides a descriptive overview of microorganism relative to SAP administration before incision/after clamping, and according to all SSI, as well as for deep wound infections and endometritis.

Five patients (0.01%) died during the 1 month follow-up. None of the deaths were associated with an SSI. Missing data analysis is reported in the Supplementary Materials.

## Discussion

### Principal findings

This largest prospective analytic study with excellent follow-up was unable to identify an additional significant risk if SAP was delayed after cord clamping. Our results show a basically unchanged SSI risk whether SAP is administered before or after clamping.

Of note, almost all estimates of the odds ratio were > 1, suggesting a numerical tendency to an increased risk in this group. Thus, the odds ratio of the main analysis does not rule out the possibility that the odds of an event may be increased by up to 36%. Many unmeasured confounders may have been present and the confidence intervals of the estimate are consistent with a potential increase in infection with administration after cord clamping.

In addition, our data show that factors other than timing of SAP administration were significantly associated with SSI risk. It was expected that increased procedure duration, increased BMI, high ASA score, contaminated wound and emergency operation were associated with an increased risk. The finding that age group of 30–40 years had a decreased risk in comparison to < 30 years may be due to a selection bias, as patients < 30 were more likely to have an emergent procedure and more frequently experienced premature rupture of the membranes. Of note, the choice of SAP agent did not play a significant role regarding SSI risk.

### Results (internal and external validity)

The internal validity of our study was excellent, as hospitals throughout Switzerland participated, including smaller institutions (< 200) and large centers (> 500) beds. The multilevel analysis approach with clustering at the hospital level allowed us to control for potential variation in SSI rate and reporting between the centers.

Concerning external validity, the analysis of large prospective registries may be the ideal source for generating high-quality scientific data [27]. The overall SSI rate was low in our study (1.7%), compared to 4–5% reported by the WHO and the meta-analyses [2, 15] and up to 6–12% from a recent randomized controlled trial [28]. The difference could be explained by the fact that former studies included patients from developing countries in a different setting [2, 15]. In the latter study, the baseline characteristics suggested a relevantly higher BMI (+ 8 kg/m2) and a higher rate of patients had already had ruptured membranes, both important SSI risk factors after cesarean delivery [28]. As this is a national multi-center study with clustering at institutional level, we are convinced that our results can be transferred to similar health-care settings in resource rich countries, but this may not be the case for lower and middle income countries.

Even though other studies have shown a decreases SSI risk for SAP before incision, this was relatively small in absolute numbers: the Cochrane review reported 38/2531 mothers developed an endomyometritis before clamping compared to 70/2510 after clamping. This potentially increased risk was not confirmed by our study and must be contrasted with a potential harm to the neonate’s immune development [5–9]. Disrupted transmission of maternal *Bacteroides* strains to babies was even seen in vaginally delivered babies whose mothers underwent antibiotic prophylaxis [9].

Finally, the potentially very short time interval between the two strategies argue against a relevant differential risk.

### Clinical implications

These results challenge the latest WHO recommendation which extends the time window of SAP from 60 min to 120 min prior to incision but does not consider administration after clamping [29].

### Research implications

In future research, the principally unchanged or slightly elevated SSI risk for the mother has to be balanced with long-term neonatal outcomes/microbiome development.

### Strengths and limitations

The main strengths of our study were the standardized evaluation of SSI cases by dedicated physicians, a post-discharge surveillance at 30 days, a mere < 10.4% loss of follow-up, routine on-site monitoring of the data collection quality, and a multilevel model that permitted adjustment for SSI variation across the institutions. The main limitation of this study, was that the exact timing of clamping was not available and therefore administration after incision served as a surrogate marker. We believe we have sufficient evidence that SAP administration after incision corresponds to “after clamping”. First, clamping is done quickly after incision, usually within 5 min [20]. This is reflected in Fig. 2, where SAP application after incision peaks between 5 and 10 min after incision, compatible with administration after clamping. Moreover, a supplementary analysis failed to identify an optimal timing window within +/− 60 min relative to incision. In addition, we performed two subgroup analyses that made post-incisional SAP administration even more likely to represent “after clamping” administration, and the results are in line with the main analysis.

Other limitations of the study were the lack of detailed information concerning the individual surgeons, type of operating room, and patient comorbidities/characteristics (such as diabetes, glycemic control, smoking, nutritional status, intraoperative core temperature, estimated blood loss, and oxygen saturation), preoperative skin preparation procedures and/or the presence of SSI intervention bundles. None of these variables are recorded in the Swissnoso database. In addition, data on antimicrobial treatment before and after surgery, group B streptococcal carriage, multiple pregnancy, and/or vaginal disinfection were not routinely recorded.

The study period extended over 10 years, with potential changes in the management of cesarean section. However, cesarean section rates in Switzerland during this period remained constant at ~ 33% [30, 31], and surgical technique has not significantly changed over this time period. Stratified analysis for the period before and after 2014 did not provide arguments for a significant confounding by the two periods.

The variable BMI was only available in 40% of patients. Increasing BMI is a well-known risk factor for SSI [28, 32, 33]. It might have been a confounding factor, but we performed several subgroup analysis with patients where BMI was available and the results were in line with the main analysis.

## Conclusions

Results of this large prospective study provide evidence that the risk of SSI for the mother in cesarean section is similar for SAP given before incision or after umbilical cord clamping.

## Supplementary Information

**Additional file 1**. Supplementary Materials.

## Data Availability

The datasets generated and analyzed during the current study are not publicly available according to Swissnoso data sharing regulations. Data is available upon reasonable request to the Swissnoso Scientific Board. (https://www.swissnoso.ch/forschung-entwicklung/reglemente/). The statistical code is available on reasonable request to the corresponding author.

## Abbreviations

WHO: The World Health Organization
SAP: Surgical antimicrobial prophylaxis
SSI: Surgical site infections
BMI: Body mass index
ASA: American Society of Anesthesiologists
CDC: Centers for Disease Control and Prevention
GAM: General additive models
OR: Odds ratio
CI: Confidence interval
