# Supplementary material for: Antimicrobial prophylaxis administration after umbilical cord clamping in cesarean section and the risk of surgical site infection: a cohort study with 55,901 patients
Source: Antimicrob Resist Infect Control. 2020 Dec 22;9:201. doi: 10.1186/s13756-020-00860-0 (PMC7754587; doi:10.1186/s13756-020-00860-0)
Supplement: Supplementary file 1 — Additional file 1. Supplementary Materials. [file 13756_2020_860_MOESM1_ESM.docx]

**Supplementary Material**

**Supplementary Table 1**

**Surgical Site Infection Rates, Stratified by Surgical Antimicrobial Prophylaxis Administration (Before incision / After clamping)**

|  | Before incision | After cord clamping | p |
| --- | --- | --- | --- |
| n | 26405 | 29496 |  |
| Overall SSI | 397 (1.6) | 449 (1.7) | 0.759 |
| Superficial wound | 247 (1.0) | 303 (1.2) | 0.236 |
| Deep wound | 34 (0.1) | 36 (0.1) | 0.953 |
| Endometritis | 116 (0.5) | 110 (0.4) | 0.278 |

Abbreviations

SSI Surgical Site Infection

**Supplementary Table 2**

**Adjusted Mixed Effects Logistic Regression Model* with Surgical Site Infection as the Dependent Variable (model among the subset of patients with data on BMI)**

|  | OR | LL | UL | p |
| --- | --- | --- | --- | --- |
| SAP administration after cord clamping (Ref= before incision) | 1.30 | 0.97 | 1.74 | 0.078 |
| Age group |  |  |  |  |
| 30-40 years (Ref= <30 years) | 0.78 | 0.62 | 0.97 | 0.026 |
| >40 years (Ref= <30 years) | 0.97 | 0.64 | 1.46 | 0.873 |
| BMI (per 1kg/m2 increase) | 1.05 | 1.03 | 1.07 | <0.001 |
| Contaminated wound (Ref= clean, contaminated) | 1.23 | 0.96 | 1.58 | 0.096 |
| SAP agent (%) |  |  |  |  |
| Cefazolin (Ref= Amoxicillin/clavulanate) | 1.06 | 0.48 | 2.32 | 0.884 |
| Ceftriaxone (Ref= Amoxicillin/clavulanate) | 1.22 | 0.52 | 2.86 | 0.645 |
| Cefuroxime (Ref= Amoxicillin/clavulanate) | 1.19 | 0.54 | 2.62 | 0.662 |
| Elective surgery (Ref= emergent) | 0.51 | 0.40 | 0.66 | <0.001 |
| ASA Score |  |  |  |  |
| ASA 2 (Ref= ASA 1) | 1.29 | 0.96 | 1.74 | 0.087 |
| ASA 3/4/5 (Ref= ASA 1) | 1.02 | 0.60 | 1.75 | 0.936 |
| Procedure duration (per 30 minutes increase) | 1.24 | 1.05 | 1.47 | 0.012 |
| Hospital bed size |  |  |  |  |
| 200-499 (Ref = <200) | 1.40 | 0.91 | 2.17 | 0.125 |
| 500+ (Ref = <200) | 1.23 | 0.73 | 2.06 | 0.433 |
| Procedure after Jan 2014 (Ref= before) | 0.95 | 0.67 | 1.35 | 0.777 |

*complete cases only, n=19,923

Abbreviations

ASA American Society of Anesthesiologists

BMI Body Mass Index

LL Lower limit of 95% confidence interval

OR Odds Ratio

SAP Surgical antimicrobial prophylaxis

UL Upper limit of 95% confidence interval

**Supplementary Table 3**

**Adjusted Mixed Effects Logistic Regression Model* with Deep Surgical Site Infection as the Dependent Variable**

|  | OR | LL | UL | p |
| --- | --- | --- | --- | --- |
| SAP administration after cord clamping (Ref= before incision) | 1.07 | 0.80 | 1.42 | 0.661 |
| Age group |  |  |  |  |
| 30-40 years (Ref= <30 years) | 0.77 | 0.61 | 0.99 | 0.039 |
| >40 years (Ref= <30 years) | 0.81 | 0.50 | 1.32 | 0.397 |
| Contaminated wound (Ref= clean, contaminated) | 1.39 | 1.05 | 1.83 | 0.023 |
| SAP agent (%) |  |  |  |  |
| Cefazolin (Ref= Amoxicillin/clavulanate) | 1.78 | 0.85 | 3.71 | 0.126 |
| Ceftriaxone (Ref= Amoxicillin/clavulanate) | 1.18 | 0.45 | 3.10 | 0.738 |
| Cefuroxime (Ref= Amoxicillin/clavulanate) | 1.71 | 0.86 | 3.42 | 0.126 |
| Elective surgery (Ref= emergent) | 0.65 | 0.49 | 0.85 | 0.002 |
| ASA Score |  |  |  |  |
| ASA 2 (Ref= ASA 1) | 1.05 | 0.77 | 1.42 | 0.770 |
| ASA 3/4/5 (Ref= ASA 1) | 1.03 | 0.59 | 1.81 | 0.911 |
| Procedure duration (per 30 minutes increase) | 1.50 | 1.25 | 1.78 | <0.001 |
| Hospital bed size |  |  |  |  |
| 200-499 (Ref = <200) | 1.35 | 0.85 | 2.16 | 0.199 |
| 500+ (Ref = <200) | 1.29 | 0.69 | 2.42 | 0.431 |
| Procedure after Jan 2014 (Ref= before) | 0.88 | 0.65 | 1.20 | 0.424 |

*complete cases only, n=48,995

Abbreviations

ASA American Society of Anesthesiologists

LL Lower limit of 95% confidence interval

OR Odds Ratio

SAP Surgical antimicrobial prophylaxis

UL Upper limit of 95% confidence interval

**Supplementary Table 4**

**Adjusted Mixed Effects Logistic Regression Model* with Superficial Surgical Site Infection as the Dependent Variable**

|  | OR | LL | UL | p |
| --- | --- | --- | --- | --- |
| SAP administration after cord clamping (Ref= before incision) | 1.16 | 0.93 | 1.44 | 0.180 |
| Age group |  |  |  |  |
| 30-40 years (Ref= <30 years) | 0.75 | 0.63 | 0.90 | 0.002 |
| >40 years (Ref= <30 years) | 0.87 | 0.62 | 1.24 | 0.447 |
| Contaminated wound (Ref= clean, contaminated) | 1.21 | 0.98 | 1.49 | 0.070 |
| SAP agent (%) |  |  |  |  |
| Cefazolin (Ref= Amoxicillin/clavulanate) | 0.87 | 0.53 | 1.42 | 0.580 |
| Ceftriaxone (Ref= Amoxicillin/clavulanate) | 0.84 | 0.46 | 1.55 | 0.578 |
| Cefuroxime (Ref= Amoxicillin/clavulanate) | 0.82 | 0.52 | 1.31 | 0.412 |
| Elective surgery (Ref= emergent) | 0.54 | 0.44 | 0.66 | <0.001 |
| ASA Score |  |  |  |  |
| ASA 2 (Ref= ASA 1) | 1.33 | 1.04 | 1.70 | 0.022 |
| ASA 3/4/5 (Ref= ASA 1) | 1.52 | 1.01 | 2.27 | 0.044 |
| Procedure duration (per 30 minutes increase) | 1.23 | 1.07 | 1.42 | 0.004 |
| Hospital bed size |  |  |  |  |
| 200-499 (Ref = <200) | 1.11 | 0.78 | 1.58 | 0.570 |
| 500+ (Ref = <200) | 1.10 | 0.69 | 1.75 | 0.677 |
| Procedure after Jan 2014 (Ref= before) | 0.85 | 0.68 | 1.06 | 0.150 |

*complete cases only, n=48,995

Abbreviations

ASA American Society of Anesthesiologists

LL Lower limit of 95% confidence interval

OR Odds Ratio

SAP Surgical antimicrobial prophylaxis

UL Upper limit of 95% confidence interval

**Supplementary Table 5**

**Adjusted Mixed Effects Logistic Regression Model* with Deep Surgical Site Infection as the Dependent Variable (model among the subset of patients with data on BMI)**

|  | OR | LL | UL | p |
| --- | --- | --- | --- | --- |
| SAP administration after cord clamping (Ref= before incision) | 1.34 | 0.86 | 2.10 | 0.197 |
| Age group |  |  |  |  |
| 30-40 years (Ref= <30 years) | 0.81 | 0.57 | 1.14 | 0.228 |
| >40 years (Ref= <30 years) | 0.89 | 0.45 | 1.75 | 0.731 |
| BMI (per 1kg/m2 increase) | 1.03 | 1.00 | 1.06 | 0.035 |
| Contaminated wound (Ref= clean, contaminated) | 1.66 | 1.12 | 2.48 | 0.012 |
| SAP agent (%) |  |  |  |  |
| Cefazolin (Ref= Amoxicillin/clavulanate) | 3.40 | 0.43 | 26.94 | 0.247 |
| Ceftriaxone (Ref= Amoxicillin/clavulanate) | 2.36 | 0.26 | 21.40 | 0.445 |
| Cefuroxime (Ref= Amoxicillin/clavulanate) | 3.55 | 0.45 | 28.22 | 0.231 |
| Elective surgery (Ref= emergent) | 0.68 | 0.45 | 1.02 | 0.064 |
| ASA Score |  |  |  |  |
| ASA 2 (Ref= ASA 1) | 1.22 | 0.77 | 1.93 | 0.408 |
| ASA 3/4/5 (Ref= ASA 1) | 1.15 | 0.50 | 2.63 | 0.746 |
| Procedure duration (per 30 minutes increase) | 1.55 | 1.21 | 1.97 | <0.001 |
| Hospital bed size |  |  |  |  |
| 200-499 (Ref = <200) | 2.30 | 1.14 | 4.63 | 0.019 |
| 500+ (Ref = <200) | 1.45 | 0.56 | 3.75 | 0.449 |
| Procedure after Jan 2014 (Ref= before) | 1.12 | 0.63 | 1.98 | 0.695 |

*complete cases only, n=19,923

Abbreviations

ASA American Society of Anesthesiologists

BMI Body Mass Index

LL Lower limit of 95% confidence interval

OR Odds Ratio

SAP Surgical antimicrobial prophylaxis

UL Upper limit of 95% confidence interval

**Supplementary Table 6**

**Adjusted Mixed Effects Logistic Regression Model* with Superficial Surgical Site Infection as the Dependent Variable (model among the subset of patients with data on BMI)**

|  | OR | LL | UL | p |
| --- | --- | --- | --- | --- |
| SAP administration after cord clamping (Ref= before incision) | 1.26 | 0.86 | 1.82 | 0.233 |
| Age group |  |  |  |  |
| 30-40 years (Ref= <30 years) | 0.76 | 0.58 | 1.01 | 0.061 |
| >40 years (Ref= <30 years) | 1.02 | 0.61 | 1.71 | 0.943 |
| BMI (per 1kg/m2 increase) | 1.06 | 1.03 | 1.08 | <0.001 |
| Contaminated wound (Ref= clean, contaminated) | 1.01 | 0.74 | 1.39 | 0.948 |
| SAP agent (%) |  |  |  |  |
| Cefazolin (Ref= Amoxicillin/clavulanate) | 0.71 | 0.29 | 1.69 | 0.435 |
| Ceftriaxone (Ref= Amoxicillin/clavulanate) | 1.03 | 0.41 | 2.59 | 0.953 |
| Cefuroxime (Ref= Amoxicillin/clavulanate) | 0.87 | 0.37 | 2.08 | 0.758 |
| Elective surgery (Ref= emergent) | 0.45 | 0.33 | 0.61 | <0.001 |
| ASA Score |  |  |  |  |
| ASA 2 (Ref= ASA 1) | 1.34 | 0.92 | 1.95 | 0.128 |
| ASA 3/4/5 (Ref= ASA 1) | 0.94 | 0.47 | 1.90 | 0.866 |
| Procedure duration (per 30 minutes increase) | 1.04 | 0.83 | 1.31 | 0.709 |
| Hospital bed size |  |  |  |  |
| 200-499 (Ref = <200) | 0.94 | 0.55 | 1.61 | 0.825 |
| 500+ (Ref = <200) | 1.00 | 0.55 | 1.82 | 0.989 |
| Procedure after Jan 2014 (Ref= before) | 0.88 | 0.58 | 1.34 | 0.549 |

*complete cases only, n=19,923

Abbreviations

ASA American Society of Anesthesiologists

BMI Body Mass Index

LL Lower limit of 95% confidence interval

OR Odds Ratio

SAP Surgical antimicrobial prophylaxis

UL Upper limit of 95% confidence interval

**Supplementary Table 7**

**Adjusted Mixed Effects Logistic Regression Model* with Surgical Site Infection as the Dependent Variable, differential timing windows**

|  | OR | LL | UL | p |
| --- | --- | --- | --- | --- |
| SAP administration Timing window (minutes) |  |  |  |  |
| -60 to -30 (Ref= -20 to -10) | 1.06 | 0.71 | 1.60 | 0.776 |
| -29 to -20 (Ref= -20 to -10) | 1.19 | 0.80 | 1.75 | 0.391 |
| -9 to -1 (Ref= -20 to -10) | 0.73 | 0.46 | 1.15 | 0.176 |
| 0 to 9 (Ref= -20 to -10) | 1.28 | 0.86 | 1.91 | 0.221 |
| 10 to 19 (Ref= -20 to -10) | 1.30 | 0.87 | 1.94 | 0.196 |
| 20 to 60 (Ref= -20 to -10) | 1.21 | 0.74 | 1.98 | 0.454 |
| Age group |  |  |  |  |
| 30-40 years (Ref= <30 years) | 0.77 | 0.62 | 0.97 | 0.024 |
| >40 years (Ref= <30 years) | 0.96 | 0.64 | 1.46 | 0.866 |
| BMI (per 1kg/m2 increase) | 1.05 | 1.03 | 1.07 | <0.001 |
| Contaminated wound (Ref= clean, contaminated) | 1.24 | 0.97 | 1.60 | 0.085 |
| SAP agent (%) |  |  |  |  |
| Cefazolin (Ref= Amoxicillin/clavulanate) | 1.08 | 0.50 | 2.33 | 0.854 |
| Ceftriaxone (Ref= Amoxicillin/clavulanate) | 1.24 | 0.54 | 2.88 | 0.613 |
| Cefuroxime (Ref= Amoxicillin/clavulanate) | 1.20 | 0.55 | 2.60 | 0.651 |
| Elective surgery (Ref= emergent) | 0.50 | 0.39 | 0.65 | <0.001 |
| ASA Score |  |  |  |  |
| ASA 2 (Ref= ASA 1) | 1.29 | 0.96 | 1.73 | 0.089 |
| ASA 3/4/5 (Ref= ASA 1) | 1.02 | 0.59 | 1.75 | 0.943 |
| Procedure duration (per 30 minutes increase) | 1.24 | 1.05 | 1.48 | 0.013 |
| Hospital bed size |  |  |  |  |
| 200-499 (Ref = <200) | 1.41 | 0.92 | 2.15 | 0.113 |
| 500+ (Ref = <200) | 1.24 | 0.75 | 2.05 | 0.410 |
| Procedure after Jan 2014 (Ref= before) | 0.96 | 0.68 | 1.35 | 0.805 |

*complete cases only, n=48,995

Abbreviations

ASA American Society of Anesthesiologists

BMI Body Mass Index

LL Lower limit of 95% confidence interval

OR Odds Ratio

SAP Surgical antimicrobial prophylaxis

UL Upper limit of 95% confidence interval

**Supplementary Table 8**

**Adjusted Mixed Effects Logistic Regression Model with Surgical Site Infection as the Dependent Variable, with administered SAP in >90% of procedures per hospital either before or after incision the adjusted OR of SSI risk**

|  | OR | LL | UL | p |
| --- | --- | --- | --- | --- |
| SAP administration after cord clamping (Ref= before incision) | 0.84 | 0.54 | 1.30 | 0.435 |
| Age group |  |  |  |  |
| 30-40 years (Ref= <30 years) | 0.84 | 0.63 | 1.13 | 0.257 |
| >40 years (Ref= <30 years) | 0.78 | 0.44 | 1.40 | 0.413 |
| Contaminated wound (Ref= clean, contaminated) | 1.30 | 0.93 | 1.81 | 0.126 |
| SAP agent (%) |  |  |  |  |
| Cefazolin (Ref= Amoxicillin/clavulanate) | 0.60 | 0.28 | 1.29 | 0.192 |
| Ceftriaxone (Ref= Amoxicillin/clavulanate) | 0.43 | 0.21 | 0.89 | 0.023 |
| Cefuroxime (Ref= Amoxicillin/clavulanate) | 0.60 | 0.30 | 1.21 | 0.154 |
| Elective surgery (Ref= emergent) | 0.56 | 0.41 | 0.78 | <0.001 |
| ASA Score |  |  |  |  |
| ASA 2 (Ref= ASA 1) | 1.11 | 0.78 | 1.57 | 0.559 |
| ASA 3/4/5 (Ref= ASA 1) | 0.97 | 0.49 | 1.91 | 0.928 |
| Procedure duration (per 30 minutes increase) | 1.02 | 0.81 | 1.29 | 0.872 |
| Hospital bed size |  |  |  |  |
| 200-499 (Ref = <200) | 1.22 | 0.82 | 1.80 | 0.326 |
| 500+ (Ref = <200) | 1.56 | 0.92 | 2.64 | 0.095 |
| Procedure after Jan 2014 (Ref= before) | 1.33 | 0.88 | 2.01 | 0.177 |

*complete cases only, n=13,614

Abbreviations

ASA American Society of Anesthesiologists

LL Lower limit of 95% confidence interval

OR Odds Ratio

SAP Surgical antimicrobial prophylaxis

UL Upper limit of 95% confidence interval

**Supplementary Table 9**

**Adjusted Mixed Effects Logistic Regression Model with Surgical Site Infection as the Dependent Variable,** **excluding the 17’205 patients that received SAP within 10min after incision**

|  | OR | LL | UL | p |
| --- | --- | --- | --- | --- |
| SAP administration after cord clamping (Ref= before incision) | 1.10 | 0.87 | 1.38 | 0.420 |
| Age group |  |  |  |  |
| 30-40 years (Ref= <30 years) | 0.80 | 0.67 | 0.96 | 0.017 |
| >40 years (Ref= <30 years) | 0.85 | 0.60 | 1.20 | 0.352 |
| Contaminated wound (Ref= clean, contaminated) | 1.27 | 1.04 | 1.56 | 0.021 |
| SAP agent (%) |  |  |  |  |
| Cefazolin (Ref= Amoxicillin/clavulanate) | 1.08 | 0.67 | 1.74 | 0.754 |
| Ceftriaxone (Ref= Amoxicillin/clavulanate) | 0.96 | 0.52 | 1.78 | 0.901 |
| Cefuroxime (Ref= Amoxicillin/clavulanate) | 0.97 | 0.62 | 1.52 | 0.898 |
| Elective surgery (Ref= emergent) | 0.58 | 0.47 | 0.70 | <0.001 |
| ASA Score |  |  |  |  |
| ASA 2 (Ref= ASA 1) | 1.15 | 0.91 | 1.46 | 0.252 |
| ASA 3/4/5 (Ref= ASA 1) | 1.38 | 0.94 | 2.03 | 0.105 |
| Procedure duration (per 30 minutes increase) | 1.34 | 1.18 | 1.53 | <0.001 |
| Hospital bed size |  |  |  |  |
| 200-499 (Ref = <200) | 1.30 | 0.96 | 1.76 | 0.092 |
| 500+ (Ref = <200) | 1.26 | 0.83 | 1.93 | 0.278 |
| Procedure after Jan 2014 (Ref= before) | 0.98 | 0.79 | 1.22 | 0.854 |

*complete cases only, n=33,935

Abbreviations

ASA American Society of Anesthesiologists

LL Lower limit of 95% confidence interval

OR Odds Ratio

SAP Surgical antimicrobial prophylaxis

UL Upper limit of 95% confidence interval

**Supplementary Table 10**

**Microbiology results (descriptive)**

|  |  | All infections | |  |  |  |  |  | Deep Infections (Wound / Endometritis) | | | | | |
| --- | --- | --- | --- | --- | --- | --- | --- | --- | --- | --- | --- | --- | --- | --- |
|  |  | Summary | | Before incision | | After clamping | |  | Summary | | Before incision | | After clamping | |
| 01_Staphylococcus aureus | Methicillin sensitive | 70 | 8.3% | 31 | 7.8% | 39 | 8.7% |  | 13 | 4.4% | 5 | 3.3% | 8 | 5.5% |
| 02_Staphylococcus aureus | Methicillin resistant | 8 | 0.9% | 4 | 1.0% | 4 | 0.9% |  | 2 | 0.7% | 1 | 0.7% | 1 | 0.7% |
| 03_Staphylococci | coagulase negative | 25 | 3.0% | 9 | 2.3% | 16 | 3.6% |  | 3 | 1.0% | 1 | 0.7% | 2 | 1.4% |
| 04_Group B Streptococci |  | 14 | 1.7% | 4 | 1.0% | 10 | 2.2% |  | 10 | 3.4% | 4 | 2.7% | 6 | 4.1% |
| 05_Other Streptococci |  | 4 | 0.5% | 2 | 0.5% | 2 | 0.4% |  | 4 | 1.4% | 2 | 1.3% | 2 | 1.4% |
| 06_Enterococci |  | 18 | 2.1% | 14 | 3.5% | 4 | 0.9% |  | 6 | 2.0% | 5 | 3.3% | 1 | 0.7% |
| 07_Other Gram positives |  | 10 | 1.2% | 5 | 1.3% | 5 | 1.1% |  | 4 | 1.4% | 2 | 1.3% | 2 | 1.4% |
| 08_Escherichia coli | non-ESBL | 23 | 2.7% | 11 | 2.8% | 12 | 2.7% |  | 21 | 7.1% | 10 | 6.7% | 11 | 7.5% |
| 09_Other Enterobacteriaceae | non-ESBL | 32 | 3.8% | 17 | 4.3% | 15 | 3.3% |  | 15 | 5.1% | 7 | 4.7% | 8 | 5.5% |
| 10_ESBL-producing Enterobacteriaceae | | 2 | 0.2% | 1 | 0.3% | 1 | 0.2% |  | 0 | 0.0% | 0 | 0.0% | 0 | 0.0% |
| 11_Pseudomonas aeruginosa |  | 5 | 0.6% | 1 | 0.3% | 4 | 0.9% |  | 0 | 0.0% | 0 | 0.0% | 0 | 0.0% |
| 12_Other non-fermenter |  | 2 | 0.2% | 1 | 0.3% | 1 | 0.2% |  | 1 | 0.3% | 1 | 0.7% | 0 | 0.0% |
| 13_Other Gram negative |  | 1 | 0.1% | 1 | 0.3% | 0 | 0.0% |  | 1 | 0.3% | 1 | 0.7% | 0 | 0.0% |
| 14_Anerobes |  | 20 | 2.4% | 11 | 2.8% | 9 | 2.0% |  | 14 | 4.7% | 9 | 6.0% | 5 | 3.4% |
| 15_Other |  | 9 | 1.1% | 6 | 1.5% | 3 | 0.7% |  | 4 | 1.4% | 2 | 1.3% | 2 | 1.4% |
| NA |  | 603 | 71.3% | 279 | 70.3% | 324 | 72.2% |  | 198 | 66.9% | 100 | 66.7% | 98 | 67.1% |

**Missing data analysis**

Several approaches were performed to estimate the impact of missing data: The variable BMI was not compulsory for SSI surveillance reporting, and reported in 40% of patients only. For the missing BMI variable, subgroup analyses (Suppl. Tables 1, 4, 5, 9) were performed that were in line with the main results.

Apart from BMI and the SSI outcome variable itself (10.7%), there were low numbers of missing baseline covariates (<1.5%; Suppl. Table 11). Regarding the missing SSI variable, we observed a worse follow-up rate in hospitals with <200 beds than in larger hospitals. After fitting the analysis model to the multiply imputed data sets assuming missing at random for all missing variables (including the outcome), the point estimates were within the 95% confidence intervals from the complete case analysis (Suppl. Table 12-13), We concluded that the results were robust to plausible assumptions concerning the missing data.

**Suppl. Table 11**

**Missing data overview**

Descriptive statistics (only those with missing data shown)

| Variable | N (%) |
| --- | --- |
| SSI |  |
| observed | 49937 ( 89.3) |
| missing | 5964 ( 10.7) |
| BMI |  |
| observed | 22339 ( 40.0) |
| missing | 33562 ( 60.0) |
| Duration of procedure |  |
| observed | 55663 ( 99.6) |
| missing | 238 ( 0.4) |
| ASA score |  |
| observed | 55081 ( 98.5) |
| missing | 820 ( 1.5) |

**Suppl. Table 12**

**Relationship between observed and missing data**

We investigated missingness by comparing those patients with missing data (i.e. one or more missing variables) with those fully observed (R = 1), with the goal to identify systematic differences.

Since there appeared to be some systematic differences, we used multiple imputation to investigate further.

|  | level | not missing any | missing any | p |
| --- | --- | --- | --- | --- |
| n |  | 48996 | 6905 |  |
| SSI (%) | 0 | 48165 (98.3) | 926 (98.4) | 0.91 |
|  | 1 | 831 ( 1.7) | 15 ( 1.6) |  |
| Timing of SAP | Before incision | 23282 (47.5) | 3123 (45.2) | <0.001 |
|  | After clamping | 25714 (52.5) | 3782 (54.8) | |
| Age Group | Age Group <30 | 14591 (29.8) | 2202 (31.9) | <0.001 |
|  | Age Group 30-40 | 30806 (62.9) | 4171 (60.4) |  |
|  | Age Group >40 | 3599 ( 7.3) | 532 ( 7.7) |  |
| Wound class (%) | clean-contaminated | 38303 (78.2) | 5434 (78.7) | 0.334 |
|  | contaminated | 10693 (21.8) | 1471 (21.3) | |
| atb1 (%) | Amoxi/Clavulanate | 4617 ( 9.4) | 754 (10.9) | <0.001 |
|  | Cefazoline | 16023 (32.7) | 2131 (30.9) | |
|  | Ceftriaxone | 5243 (10.7) | 656 ( 9.5) |  |
|  | Cefuroxime | 23113 (47.2) | 3364 (48.7) |  |
| elective (%) | No | 24111 (49.2) | 3512 (50.9) | 0.011 |
|  | Yes | 24885 (50.8) | 3393 (49.1) | |
| ASA Score | ASA 1 | 10052 (20.5) | 1237 (20.3) | 0.005 |
|  | ASA 2 | 36405 (74.3) | 4472 (73.5) |  |
|  | ASA 3/4/ | 2539 ( 5.2) | 376 ( 6.2) | |
| Duration (minutes) | 0-14 | 993 ( 2.0) | 176 ( 2.6) | <0.001 |
|  | 15-44 | 31305 (63.9) | 4469 (67.0) | |
|  | 45-74 | 15217 (31.1) | 1858 (27.9) |  |
|  | 75-104 | 1216 ( 2.5) | 145 ( 2.2) |  |
|  | 105-134 | 235 ( 0.5) | 15 ( 0.2) |  |
|  | >135 | 30 ( 0.1) | 4 ( 0.1) |  |
| hosp_size (%) | <200 | 25678 (52.4) | 4238 (61.4) | <0.001 |
|  | 200-499 | 15419 (31.5) | 1835 (26.6) |  |
|  | 500+ | 7899 (16.1) | 832 (12.0) |  |
| year (%) | before 1.1.2014 | 24984 (51.0) | 3224 (46.7) | <0.001 |
|  | after 1.1.2014 | 24012 (49.0) | 3681 (53.3) |  |

**Suppl. Table 13**

**Comparison of estimates from the complete case analysis and following multiple imputation assuming missing at random**

We employed multiple imputation to impute values with missingness. We used K = 50 imputed data sets using the MICE package in R, including all covariates and the outcome in the imputation model. We note that for categorical variables MICE uses polytomous logistic regression to impute missing values i.e. multinomial logistic regression.

|  | **Complete Case analysis** | | | **Assuming Missing at Random** | | |
| --- | --- | --- | --- | --- | --- | --- |
|  | OR | L95 | U95 | OR | L95 | U95 |
| SAP administration after cord clamping (Ref= before incision) | 1.05 | 0.90 | 1.23 | 1.05 | 0.90 | 1.22 |
| Age group |  |  |  |  |  |  |
| 30-40 years (Ref= <30 years) | 0.76 | 0.66 | 0.88 | 0.75 | 0.64 | 0.86 |
| >40 years (Ref= <30 years) | 0.84 | 0.63 | 1.11 | 0.82 | 0.61 | 1.08 |
| Contaminated wound (Ref= clean, contaminated) |  |  |  |  |  |  |
| SAP agent (%) | 1.09 | 0.83 | 1.42 | 1.07 | 0.82 | 1.41 |
| Cefazolin (Ref= Amoxicillin/clavulanate) | 0.91 | 0.64 | 1.29 | 0.88 | 0.62 | 1.26 |
| Ceftriaxone (Ref= Amoxicillin/clavulanate) | 1.14 | 0.89 | 1.47 | 1.14 | 0.88 | 1.48 |
| Cefuroxime (Ref= Amoxicillin/clavulanate) | 0.55 | 0.46 | 0.66 | 0.57 | 0.48 | 0.67 |
| Elective surgery (Ref= emergent) |  |  |  |  |  |  |
| ASA Score | 1.23 | 1.02 | 1.49 | 1.26 | 1.05 | 1.52 |
| ASA 2 (Ref= ASA 1) | 1.27 | 0.92 | 1.77 | 1.32 | 0.94 | 1.82 |
| ASA 3/4/5 (Ref= ASA 1) | 1.31 | 1.08 | 1.59 | 1.30 | 1.10 | 1.54 |
| Procedure duration (per 30 minutes increase) | 1.36 | 1.21 | 1.52 | 1.33 | 1.20 | 1.48 |
| Hospital bed size |  |  |  |  |  |  |
| 200-499 (Ref = <200) | 1.20 | 1.01 | 1.42 | 1.20 | 1.02 | 1.42 |
| 500+ (Ref = <200) | 1.24 | 1.00 | 1.54 | 1.27 | 1.02 | 1.58 |
| Procedure after Jan 2014 (Ref= before) | 0.91 | 0.77 | 1.07 | 0.90 | 0.76 | 1.06 |
